# The Emergence and Development of Bioethics in the Uk

**DOI:** 10.1093/medlaw/fwy011

**Published:** 2018-04-04

**Authors:** Ruth Chadwick, Duncan Wilson

**Affiliations:** 1 Centre for Social Ethics and Policy (CSEP), University of Manchester, M13 9PL, UK; 2 Centre for the History of Science, Technology and Medicine (CHSTM), University of Manchester, M13 9PL, UK

**Keywords:** Bioethics, History, Methods

## Abstract

Bioethics emerged in a specific social and historical context. Its relationship to older traditions in medical ethics and to environmental ethics is an ongoing matter of debate. This article analyses the social, institutional, and economic factors that led to the development of bioethics in the UK in the 1980s, and the course it has taken since. We show how phenomena such as globalisation, the focus on ‘ethical legal and social issues’ and the empirical turn have affected the methods employed, and argue that ongoing controversies about the nature and possibility of ethical expertise will affect its future.

## I. INTRODUCTION

Written by a historian and a bioethicist, this article presents an overview of the emergence and development of bioethics in the UK since the 1980s. It is by no means comprehensive, but reflects our perspective after years working on and in bioethics. We believe it provides important context for the topics discussed in other articles in this special issue; not least by helping us reflect on why academics from philosophy, law, and the social sciences began to discuss and help regulate matters that had long been the preserve of doctors and scientists. We aim to identify some key trends without purporting to offer a detailed history.

Derived from the Greek words *bios* (life) and *ethike* (ethics), ‘bioethics’ is one of the most recognisable neologisms of recent decades. The term initially denoted an approach few of us would recognise today. During the 1920s the German pastor Fritz Jahr defined ‘bio-ethik’ as the assumption of more compassionate attitudes towards animals and plants based on scientific research that showed commonalities across species barriers.^1^ Unaware of Jahr’s work, claiming the term came to him ‘with a Eureka feeling’, in 1970, the American biochemist Van Rensselaer Potter characterised bioethics as a new ‘science of survival’ that drew on ecology and biomedical science in order to underpin decision-making in the face of a looming environmental crisis.^2^ In contrast to Jahr, who sought to extend moral consideration to non-humans, Potter viewed bioethics as an anthropocentric system of ethics designed to secure ‘the future of earth’s biological resources for human needs’.^3^

Independently of Potter, the Dutch obstetrician André Hellegers and the political activist Sargent Shriver also coined the term ‘bioethics’ in 1970 when they opened the Joseph and Rose Kennedy Institute for the Study of Human Reproduction and Bioethics at Georgetown University, a private Jesuit institution in Washington DC.^4^ Hellegers and Shriver’s definition of bioethics is the one we recognise today. Amid growing discussion of the social impact of biological research, the rationing of new medical technologies such as kidney dialysis and the rights of patients and experimental subjects, they viewed bioethics as the scrutiny of ethical issues raised by medicine and the biological sciences. This definition quickly rose to prominence. Between 1972 and 1974, the theologian Warren Reich began work on an *Encyclopedia of Bioethics*, the philosopher Daniel Callahan wrote an article on ‘Bioethics as a Discipline’ and the Library of Congress adopted ‘bioethics’ as a subject heading.^5^ ‘Bioethics’ in all these instances focused on advances in biomedical research and clinical practice, not on issues associated with ecology or environmental science.

The focus on medical practice and research appeared to continue a long-standing tradition that had been labelled as ‘medical ethics’ since the early 19th century, but bioethics differed in one crucial respect. In the USA and elsewhere, medical ethics had long been considered a matter for doctors. Discussion of ethics was confined to professional books and regulatory codes, and few people questioned whether doctors were best placed to determine what constituted good conduct in their own field.^6^ When lawyers and religious figures engaged with medical ethics during the 19th century and for much of the 20th century, they sought to consolidate the authority of doctors by clarifying the legal and ethical aspects of contentious issues such as abortion.^7^ Pointing to declining confidence in professions during the 1960s and 1970s, caused in part by the growth of radical politics, the exposure of unethical experiments on vulnerable populations and concerns over dilemmas raised by new procedures such as organ transplantation, advocates of bioethics claimed this paternalistic stance had become untenable. Callahan argued that people no longer believed ‘a good training in medicine’ led to ‘good ethical decisions’, and concluded that lawyers, philosophers, theologians, and others should now play an active role in drawing up codes of conduct for medicine and the biological sciences.^8^

This argument appealed to doctors and scientists concerned by public criticism of medical research, and to academics in fields such as philosophy who were keen to utilise their training ‘in a more applied way’.^9^ It also appealed to politicians such as Senator Edward Kennedy, who argued that federal policy should not emanate ‘just from the medical profession, but from the ethicists, the theologians, the lawyers and many other disciplines’.^10^ Kennedy was instrumental in persuading President Nixon to establish a National Commission for the Protection of Human Subjects in Biomedical and Behavioural Research; and the act that established this commission notably stipulated that no more than five of its 11 members should be scientists or doctors, with the majority drawn from law, philosophy, theology, social sciences, and the general public.^11^ In 1978, the commission issued binding guidelines for experiments involving human subjects in the Belmont Report, which ruled that all researchers should adhere to three core principles of respect for persons, beneficence, and justice. This principles-based approach, outlined in detail by Tom Beauchamp and James Childress’s influential 1979 book *The Principles of Biomedical Ethics*, ‘set out a clear and simple statement of the ethical basis of research’ and quickly became the dominant framework in American bioethics.^12^ By the turn of the 1980s, as the historian David Rothman remarks, ‘it was clear that the monopoly of the medical profession in medical ethics was over. The issues were now public and national – the province of an extraordinary variety of outsiders’.^13^

Although this definition of bioethics emerged in the USA, it soon became a global phenomenon. Members of several disciplines now scrutinise ethical issues and help regulate the conduct of doctors and biomedical scientists across Europe, in Australia, Canada, Israel, Latin America, Japan, Pakistan, Singapore, and South Korea.^14^ As we scrutinise bioethics in these locations it becomes clear that we cannot generalise from its history in the USA. The sociologist David Reubi, for example, shows how the development of bioethics in Singapore during the 1990s owed little to radical politics or the exposure of unethical research, but was part of state efforts to encourage foreign investment in biomedicine. Politicians, Reubi argues, viewed bioethics as central to reassuring incoming scientists and companies that Singapore had rigorous ethical standards and was a safe place to invest.^15^

These findings prevent us from mistakenly viewing bioethics as a monolithic entity with a universal history, and encourage us to recognise instead that what count as ‘bioethical’ problems, approaches and solutions differ across specific times and places. This was certainly the case in the UK. Despite public criticism of medical research in the 1960s and 1970s, politicians believed the best solution here was for ‘the medical profession to get its house in order’, while the *British Medical Journal* labelled bioethics ‘an American trend’.^16^ By the 1980s and 1990s, however, members of several professions began to play a leading role in developing laws for new procedures such as *in vitro* fertilisation (IVF) and embryo research; students increasingly learnt about ethical issues in medicine not from doctors but from philosophers and lawyers, who often worked in new academic centres for medical law and bioethics; interdisciplinary journals considered problems that were previously confined to medical publications; and newspapers portrayed a growing number of philosophers, lawyers, and theologians as ‘ethics experts’ whose input was central to debates concerning medicine and the biological sciences.^17^

In this article, we detail how bioethics emerged as a high profile and valued approach in the UK thanks to the interplay between changing political agendas and institutional, professional and personal concerns. We argue that a significant factor in the development of UK bioethics was that politicians in the 1980s and 1990s no longer believed medical researchers should be solely responsible for discussing and resolving ethical questions that arose in the course of their work. From the 1979 election onwards, members of successive conservative and ‘New Labour’ governments argued that professions should be exposed to outside scrutiny in order to make them publicly accountable. This political shift benefitted individuals who promoted bioethics for different reasons, including the academic lawyer Ian Kennedy, an advocate of civil rights politics who argued it was vital to democratising medicine, and philosophers such as Mary Warnock, among others, who believed engagement with practical issues would make their field relevant. The development of bioethics also stemmed from the way in which early bioethicists presented their work as a vital intermediary: claiming outside involvement with ethical decision-making would ‘reduce the burden of responsibility’ on doctors and scientists whilst reassuring politicians and the public that ‘no nameless horrors were going on in laboratories’.^18^ This argument resonated with healthcare professionals who acknowledged ‘the era which required paternalism is past’, and journals that dismissed bioethics as ‘an American trend’ in the 1970s now portrayed it as vital to ensuring ‘scientific progress’.^19^

We also detail how UK bioethics was generally regarded less as a stable discipline and more as what Onora O’Neill calls ‘a meeting ground for a different number of disciplines, discourses and organisations’.^20^ Opinions regarding appropriate methods and solutions remained divided within as well as between the disciplines that constituted this new ‘meeting ground’. This was evident in debates concerning whether or not bioethicists were moral experts who could foster agreement on difficult ethical issues, and in more recent discussions about the benefits and drawbacks of empirical, global, communitarian, and feminist ‘turns’ in bioethics. Yet, these differences of opinion did not prevent the continued growth of bioethics, evidenced by the formation of several academic centres in UK universities throughout the 1980s and 1990s, and the national Nuffield Council for Bioethics in 1991. Nor did they shake the enthusiasm for bioethics on the part of politicians and the media, with the BBC nominating Mary Warnock as one of the most influential people of the 1980s, the Labour government knighting Ian Kennedy for ‘services to bioethics’ in 2001 and the *Independent* newspaper selecting the philosopher John Harris as one of the UK’s most influential thinkers in 2006.^21^ With this in mind, we argue the continued appetite for bioethics can best be explained by viewing it as what Bill Readings calls a ‘*community of dissensus*’: where a lack of consensus is productive because, to quote the sociologist Les Back, ‘it drives us to think harder about the key issues and problems of our time’.^22^

## II. THE EMERGENCE OF BIOETHICS IN REGULATORY COMMITTEES, PUBLIC DEBATES AND UK UNIVERSITIES IN THE 1980s AND 1990s

During the 19th century British doctors viewed medical ethics as an internal concern that functioned as what the historian Harold Perkin calls a ‘strategy of closure’.^23^ It helped doctors consolidate their professional expertise by limiting disputes, excluding unqualified practitioners and allowing them to position themselves as the only group capable of providing an essential service. Thomas Percival’s 1803 book *Medical Ethics*, for instance, asserted the need for cordial relations and self-regulation among orthodox doctors to maintain the support of patients who could just as easily choose the services of alternative therapists such as homeopathists or bonesetters.^24^ To Percival and the medical reformers he influenced in the mid-19th century, any discussion of medical ethics should be produced by doctors and for doctors. This argument resonated with Victorian *laissez-faire* attitudes towards regulation, and the 1858 Medical Act officially granted doctors ‘self-governing authority’ by leaving them in charge of the new General Medical Council (GMC) that controlled registration, education, and discipline.^25^

This situation persisted well into the 20th century. When Clement Attlee’s Labour government sought to implement its 1946 National Health Service Act, doctors agreed to reform on the condition there would be as little scrutiny as possible of their ‘privileged clinical position or research practices’.^26^ Support for self-regulation was strengthened during the 1950s thanks to advances such as effective anti-tuberculosis drugs, open-heart surgery, kidney transplants and the discovery of DNA’s helical structure. Many doctors and scientists hailed these projects as evidence of the benefits of professional freedom, and celebratory press coverage portrayed them as pioneering figures who were central to a ‘new Elizabethan’ era of progress and discovery.^27^

But simply focusing on the arguments of doctors or medical researchers cannot tell the whole story. As the sociologist Andrew Abbott argues, professions do not emerge or develop in isolation and we need to move from ‘an individualistic to a systematic view’.^28^ We cannot fully appreciate the persistence of the belief that ethics was an internal concern without also studying the ‘hands off’ approach other professions adopted when they considered medical practice and research. The decisions in two medical negligence cases from the 1950s demonstrate how lawyers and judges believed, like doctors and politicians, that ‘the medical profession should be held in special regard and interfered with as little as possible’.^29^ The first case, *Hatcher v Black*, arose after a patient claimed they were not informed about possible nerve damage during thyroid surgery. Ruling in favour of the doctors, the judge, Denning J, warned that giving courts the power to decide what constituted negligent behaviour would lead to ‘defensive medicine’ where doctors thought ‘more of their own safety than the good of their patients’.^30^ The second case, *Bolam v Friern Hospital Management Committee*, arose in 1957 when a patient sued doctors for injuries that arose after they failed to restrain him during electroconvulsive therapy and did not warn him of the risks beforehand.^31^ Here, as in *Hatcher v Black*, the judge ruled in favour of the doctors. Their decision hinged not on the possibility of ‘defensive medicine’ but on the argument that the patient’s treatment conformed to standard medical practice. This ruling became known as the ‘*Bolam* test’ and was applied to virtually all medical negligence cases, until the UK Supreme Court’s 2015 ruling in *Montgomery v Lanarkshire Health Board*^32^ established that, in the context of informed consent, a patient should be told whatever they would want to know about the nature and risks of medical procedures, and not simply ‘what the doctor thinks they should be told’.^33^ As Margaret Brazier notes, by deciding that medical conduct should be judged according to professional norms, not the expectations of patients or the public, the underlying presumption in the courts for nearly 60 years ‘was that “doctor knew best”’.^34^

Philosophers adopted a similar stance, albeit for different reasons. In his influential 1903 book *Principia Ethica*, G E Moore argued that notions of ‘good’ so central to moral philosophy did not refer to a natural property and that we could not prove an action was good in the same way that, for example, we can demonstrate blood flows around the body.^35^ In his iconoclastic 1936 book *Language Truth and Logic,* A J Ayer drew on Moore’s argument and the logical positivism of the Vienna circle to portray moral statements as simply ‘expressions of emotion that can be neither true nor false’.^36^ To say a course of action was right or wrong, in effect, amounted to little more than saying ‘Hurrah!’ or ‘Boo!’.^37^ Ayer claimed that since philosophers should only scrutinise verifiable propositions, ‘a strictly philosophical treatise on ethics should make no ethical pronouncements’.^38^ His work had a lasting effect on mid-20th century UK philosophy, and on the rare occasions that philosophers responded to the ethical work of doctors and scientists, it was to reaffirm why they avoided normative issues. When the biologist Conrad Waddington told Ludwig Wittgenstein he was writing an essay for *Nature* on ‘science and ethics’ in 1942, the horrified philosopher replied it ‘was a terrible business – just terrible! You can at best stammer when you talk of it’.^39^ C E M Joad was the only philosopher who publicly responded to Waddington’s essay, but this was only to chide him for presuming that notions such as ‘good’ could be easily identified.^40^

This collective ‘hands off’ attitude was evident following the 1967 publication of *Human Guinea Pigs* by the medical whistleblower Maurice Pappworth, who outlined how NHS patients had been unwittingly exposed to unnecessary and dangerous procedures, such as cardiac catheterisation, as part of medical research. Pappworth claimed that in order to prevent future ‘dangers and indignity’, it was essential that ‘our laws do not place the entire authority to decide what is permissible and what is not in the hands of one professional class’.^41^ He argued that medical ethics should no longer be considered a matter for doctors alone, and urged the government to pass a law requiring all research projects to be scrutinised by a ‘consultation committee’ that contained at least one outsider, ‘preferably but not essentially a lawyer’.^42^ Despite favourable media coverage of Pappworth’s work, the majority of politicians continued to endorse *laissez-faire* attitudes to regulation. Members of Harold Wilson’s Labour government, elected on a promise to turn the ‘white heat’ of science and technology into economic prosperity, were reluctant to interfere with medical expertise and reiterated that ethical questions were ‘for the profession to consider’.^43^ The lawyer Cecil Clothier, meanwhile, drew on the ruling in *Hatcher v Black* when he wrote to Pappworth rejecting calls for statutory oversight. Clothier argued that formal scrutiny was inappropriate when doctors were faced with severely ill patients whose only chance of survival ‘could include trying a newly-devised drug if nothing else had done any good’.^44^ Fear of litigation and criminal prosecution might prevent doctors from trying experimental procedures in such cases, he concluded, and ‘individual assessment’ remained the best form of governance.^45^

In contrast to lawyers, philosophers and politicians, growing numbers of religious figures began to endorse what the Cambridge theologian Ian Ramsey called ‘trans-disciplinary’ involvement with medical ethics during the 1960s. There were obvious professional motivations behind their argument. Attendance at Sunday school, Protestant churches and religious rites of passages fell away dramatically in the 1960s, and a young generation were less concerned with ethics surrounding faith, God and the afterlife than with the environment, gender, nuclear weapons, and political activism.^46^ Ramsey argued it was only by placing itself within interdisciplinary discussion of contemporary issues, including ‘medical moral problems’, that theology ‘may find a new prospect and a new relevance’.^47^ He was also clear that interdisciplinary involvement with ethical issues would benefit doctors, helping reconcile them to the problems of increasingly secular and ‘pluralist societies’ where there was no longer agreement on what constituted a right course of action.^48^

Crucially, and in contrast to bioethicists in the USA, Ramsey reassured doctors that input from theologians, philosophers, and others did ‘not in any way compromise the surgeon’s or physician’s responsibility for making decisions’, but was simply intended to facilitate ‘responsible debate’ and help them better understand the moral implications of issues such as organ transplantation or IVF.^49^ Ramsey’s argument was endorsed by the first editor of a new interdisciplinary *Journal of Medical Ethics*, the theologian and philosopher Alastair Campbell, who claimed in 1976 that it aimed to help doctors make ‘more informed decisions’. Despite the involvement of other professions in discussing medical ethics, Campbell claimed the final decisions ‘remain medical ones and the responsibility remains with that profession’.^50^

While theologians were central to redefining medical ethics as a ‘trans-disciplinary’ endeavour in the UK, it was lawyers who went a step further from the late 1970s onwards and began to demand that members of other professions should play an active role in determining what constituted good professional conduct. These calls were led by Ian Kennedy, who notably labelled this more interventionist approach ‘bioethics’.^51^ Influenced by civil rights politics in the 1960s and 1970s, Kennedy believed that professions should ‘respect each person’s autonomy, his power to reach his own decisions and act on them’.^52^ After encountering bioethics during a spell teaching in the USA during the early 1970s, he claimed to find ‘much of value’ in the work of the lawyers, philosophers, and religious figures who endorsed outside involvement with medical decision-making.^53^ On returning to the UK he argued that discussion of medical ethics here was ‘too narrow’ and criticised lawyers, politicians, and others for ‘saying these are medical matters and shifting responsibility for decisions back to the hapless doctor’.^54^ In journal articles and several documentaries for BBC radio, on subjects such as withdrawing treatment from patients with no hope of recovery, Kennedy claimed that doctors and medical scientists ‘function within a framework of legal and social rules that go beyond the rules of their particular profession and must be observed’.^55^ Like the American bioethicists whose ‘brilliant insights’ he praised, Kennedy believed the solution was for ‘all interested parties’ to have a say in developing codes of practice for new or publicly contentious procedures.^56^

Kennedy discussed these proposals in detail during his 1980 BBC Reith Lectures, broadcast with the provocative title *Unmasking Medicine*. The major thrust of the six lectures was that standards for doctors and medical scientists ‘will have to be set by others, and the principle of outside scrutiny, a key feature of consumerism, seems inevitable’.^57^ This was especially the case with teaching ethics to medical students, which Kennedy argued should be central to the curriculum and undertaken ‘not by some superannuated elder statesman nor by the latest star in the medical firmament, but by an outsider, someone who is not deafened by the rhetoric of medicine’.^58^

The seemingly confrontational tone of these proposals led some to dismiss Kennedy’s lectures as ‘doctor bashing’.^59^ But he again emulated American bioethicists such as the Yale lawyer Jay Katz, who promised not to ‘indict or stifle research’, by portraying outside involvement as a help rather than a hindrance.^60^ He argued lawyers, philosophers, and others were trained to scrutinise ethical issues and that when confronted by particular dilemmas ‘it may be the doctor who is the layman’.^61^ Bioethics would, therefore, provide ‘great help to doctors in that it offers a guide to what they need to do where none existed before’.^62^ Kennedy reassured doctors that he wanted to establish ‘a relationship of partners in the enterprise of health’, in which outsiders were ‘not interfering but trying to help’.^63^

Several commentators pointed out that Kennedy’s promotion of bioethics resembled Maurice Pappworth’s calls for outside involvement in the regulation of medical research.^64^ Yet while Pappworth’s proposals were dismissed in the 1960s, senior doctors were far more receptive to Kennedy’s arguments in the 1980s. This change can be explained by the shifting political landscape that followed the election of Margaret Thatcher’s Conservative Party in 1979. Thatcher’s government lauded private enterprise and regarded state-supported and self-regulating professions as unresponsive to the entrepreneurial outlook they saw as vital to regenerating the country. Their solution, as Nigel Lawson set out in 1980, was to remodel professions on market lines; and throughout the 1980s, in cases such as teaching, local government and social services, reliance on professional expertise gave way to forms of outside scrutiny that were designed to ensure transparency, value-for-money and accountability to end users who were increasingly viewed as ‘consumers’.^65^

Ian Kennedy’s political background ensured he was no fan of the conservative government, and he often criticised its neo-liberal belief that many aspects of public life ‘could be regulated (if that is the right word) entirely by market forces’.^66^ But his demands for outside involvement and patient empowerment nevertheless mapped onto the government’s desire for publicly accountable and ‘customer focussed’ professions. This was not lost on doctors. John D Swales, head of the University of Leicester’s medical school, acknowledged Kennedy’s ‘views enjoy the enormous advantage of following the current political tide’ and recommended that ‘doctors should look closely at what he is saying’.^67^ Sir Douglas Black, President of the Royal College of Physicians, similarly believed that

Kennedy’s views have to be taken seriously, both for their own sake and because they are representative of the forces that seek to effect a radical change in the focus of medicine.^68^

The changing ‘political tide’ was evident in 1982, when the government responded to growing press disquiet surrounding the ‘aberrations of the baby revolution’ by announcing a public inquiry into IVF and embryo research.^69^ In a break from long-standing reliance on scientific or medical expertise, figures at the Department for Health and Social Security prioritised the appointment of an ‘outside chairman’.^70^ The government’s decision to appoint the philosopher Mary Warnock as head of an inquiry where members of various professions outnumbered doctors and scientists was notably praised by Ian Kennedy as ‘evidence that progress along the lines I advocate has recently been made’.^71^ Like Kennedy and members of the government, Warnock presented outside scrutiny as vital to ensuring public accountability. Writing for the popular *New Scientist* magazine in 1984, she argued that when medical research raised a moral dilemma, there was

no reason why scientists should be responsible by themselves for solving it … Increasingly, and rightly, people who are experts expect, as of right, to help determine what is or is not a tolerable society to live in.^72^

Warnock also presented outside scrutiny as beneficial to scientists and doctors. She claimed it would safeguard public and political trust by ensuring. She also argued it would safeguard public and political trust by ensuring ‘that no nameless horrors are going on in laboratories’, which would allow researchers ‘to get on with their work, without the fear of private prosecution or disruption by those who object to what they are doing’.^73^

Like Kennedy, Warnock promoted outside scrutiny of biomedical research for specific reasons. She was one of a growing number of philosophers who believed the mid-century reluctance to engage with practical issues had rendered the field irrelevant. In a 1960 book on *Ethics Since 1900*, Warnock complained that philosophy had for too long been characterised by ‘the refusal of philosophers to commit themselves to moral opinions’.^74^ But she closed the book on an optimistic note by claiming ‘the most boring days were over’.^75^ Warnock drew here on the work of Philippa Foot, who wrote a 1958 article seeking to counter Moore’s ‘naturalistic fallacy’ by arguing that moral statements could not be separated from the benefits or harms they produced in specific contexts.^76^ To Warnock, Foot’s work allowed philosophers to focus on ‘both description of the complexities of actual choices and actual decisions, and also discussion of what would count as reasons for making this or that decision’.^77^ A new generation of philosophers, initially based in Oxford, now began to pursue what Peter Singer called ‘applied ethics’ and worked on the morality of arguments relating to acts and omissions, civil disobedience, and political violence.^78^

In a 1978 edition of *Ethics Since 1900*, Warnock argued this approach was vital if philosophy was to become ‘a practical subject and therefore more urgent and interesting’.^79^ Other philosophers, in turn, believed Warnock’s role as chair of the government inquiry into IVF and embryo research demonstrated the value of ‘applied ethics’, even if they disagreed with her committee’s policy recommendations. To Singer, for example, her appointment showed how ‘the broader community has willingly accepted the relevance and value of philosophers to practical issues’, which was ‘particularly notable in bioethics’.^80^

Other philosophers were prompted to assert the value of practical approaches after the government cut the block grant it distributed to universities through the University Grants Commission (UGC) in 1981. The government announced that reductions were to be imposed selectively between institutions and subject areas; and given the government’s emphasis on meeting ‘national needs’ and enthusiasm for commercial approaches, academics rightly predicted the UGC would prioritise disciplines that were seen to contribute to economic growth, while penalising those they viewed as unproductive.^81^ Letters sent to each university advised Vice-Chancellors to protect ‘big science’ from budget cuts and ‘downgrade the arts’.^82^ Senior academics in fields such as philosophy were encouraged to take early retirement and were not replaced, making it easier for politicians and administrators to criticise shrinking departments as ‘weak and ineffectual’. These pressures were compounded in 1988, when a new Universities Funding Council announced plans to distribute money based on new ‘research assessment exercises’ that judged the ‘quality’ of a department’s research according levels of grant income and journal publications.

Many academics in arts and humanities departments recognised that these new criteria favoured the sciences and engineering, and believed they stood a better chance of gaining funding and meeting expectations that research had to confer ‘social benefits’ if they worked in areas with practical relevance.^83^ Some academics and university managers also argued it was ‘possible to improve both performance and image by casting down old-fashioned departmental barriers and abandoning worn-out subject divisions’.^84^ This combination of factors prompted growing numbers of academics to assert the value of bioethics during the 1980s and 1990s. Bioethics appealed to staff in the humanities who sought funding for applied work, and its presentation as a ‘partnership’ made it an obvious subject for interdisciplinary collaboration.^85^

While budget cuts were not their sole motivation, academics keen to work with like-minded colleagues in other disciplines began to promote outside involvement in teaching medical ethics to senior figures in university medical schools. Their efforts received support from a 1987 Institute of Medical Ethics report and the GMC’s 1993 report, *Tomorrow’s Doctors*, which both recommended that ethics should be central to the medical curriculum and presented input from a variety of perspectives as important to giving students ‘a clear grasp of the issues involved’.^86^ With medical students often demanding that more time be spent discussing ethics, senior doctors welcomed outside involvement as a ‘splendid idea’.^87^ Growing numbers of philosophers, lawyers, and others subsequently taught ethics to medical students and established new postgraduate degrees aimed primarily at healthcare professionals. Keen to formalise their collaborative work, the academics who taught on these new degrees also began to establish centres dedicated to research and teaching in bioethics and medical law; and by the late 1990s, these new centres brought together individuals from different fields at Bristol, Cardiff, Edinburgh, Glasgow, Keele, King’s College London, Liverpool, Manchester, Newcastle, Oxford, Nottingham, Preston, and Swansea. Many of the new centres received praise from university managers as they secured postgraduate fees and grant income from external funding bodies such as the European Commission and the Wellcome Trust: helping academics in philosophy, law and, later, the social sciences assert the value of their work in an increasingly austere and competitive climate.^88^

## III. METHODS IN BIOETHICS

The extent to which national factors shaped UK bioethics is also apparent when we survey the methods bioethicists employed and endorsed in their work. With notable exceptions such as the physician and philosopher Raanan Gillon, who replaced Alastair Campbell as editor of the *Journal of Medical Ethics*, prominent UK bioethicists largely rejected the principles-based approach endorsed by the majority of their counterparts in the USA.^89^ There was little agreement, however, on which methods should take precedence. While some believed that bioethicists with a philosophical background were ‘moral experts’ or ‘specialists in ethics’, who could foster consensus by providing a framework for analysing specific issues, others argued that adherence to a particular theory could not capture the range of viewpoints held in pluralist societies and was likely to leave ‘people more dogmatic or muddled than before’.^90^ The handbook for a module at the University of Manchester’s Centre for Social Ethics and Policy, which was established in 1987, embodied this latter viewpoint when it argued that the value of bioethics did ‘not lie in its ability to provide answers…to the difficult problems faced by healthcare professionals and others’, but lay instead ‘in its ability, first, to widen awareness of the issues involved and sensitivity to them; secondly to clarify one’s thinking about these issues’.^91^

But there were also broad similarities between the US and the UK. During the 1980s the disciplines that constituted bioethics on both sides of the Atlantic were primarily law and ‘applied ethics’, with theologians involved to a lesser extent. Even though many social scientists worked on issues such as IVF and organ transplantation, the majority shied away from engaging with bioethics and instead criticised it for what they saw as a ‘tendency to distance and abstract itself from the human settings in which ethical questions are embedded and experienced’.^92^ Some philosophers and lawyers took offence at this negative characterisation of their work, viewing the social scientist as ‘the team member who does nothing to help but only criticizes team performance’, and relations remained ‘tentative, distant and susceptible to strain’ throughout the 1980s.^93^

This changed during the 1990s, however, as social scientists began to outline how bioethics might benefit from sociological or ethnographic perspectives. Motivated in part by the continued demand for practically oriented work, UK sociologists argued that a more ‘bottom up’ approach could help connect bioethics to the actual expectations of doctors and patients, who often displayed preferences, values, and forms of reasoning different to those prioritised in bioethical texts.^94^ Their arguments were well received by many lawyers and philosophers who worked in university centres and the Nuffield Council of Bioethics; and as social scientists published in bioethics journals and helped determine public policy, many began to talk of an ‘empirical turn’ in bioethics.^95^ By the 21st century, social scientists joined colleagues from law and philosophy in describing bioethics as a ‘dynamic, changing, multi-sited field’, where individuals from a growing number of disciplines ‘claim the title of bioethicists’.^96^

This development fostered a new research agenda that scrutinised the ways in which the disciplines that constitute bioethics can and should relate, addressing questions such as the extent to which theoretical reflection of a philosophical sort can be integrated with empirical work done by sociologists, anthropologists, and psychologists. It was by no means the case that all scholars working in the field wanted to work in a multidisciplinary way and disagreement continued over the role of empirical evidence in bioethics; and while some took the view that integration was possible and desirable, differences remained over what form integration should take. What was called ‘integrative bioethics’ suggested the emergence of a new discipline.^97^ Advocates of ‘integrated bioethics’,^98^ on the other hand, called for a deep and continual interaction between the constituent disciplines, while others argued that the disciplines needed to maintain their distinctive methodologies and distance, working together in a ‘complementary’ way.^99^ These debates, along with attempts to define the boundaries of bioethics, continue.

Stemming in part from the reaction to issues such as genetically modified food, the empirical turn was also marked by an increasing focus on public involvement with bioethics. While it might be argued that this was not a part of bioethics *per se*, public participation and ‘engagement’ was something that bioethicists had to consider in both their funding proposals and outputs. Another change, again related to the relationship between bioethics and different funding agendas, was the ‘ELSI-fication’ of work from the 1990s onwards.

ELSI emerged after a proportion of the Human Genome Project budget in the USA was set aside to investigate ‘ethical, legal and social issues (ELSA)’. Other countries quickly followed suit. In Europe, for instance, ELSA, designating a focus on ‘ethical, legal and social *aspects*’, was the acronym of choice. The choice of ‘aspects’ over ‘issues’ provided greater scope for input from social scientists, in offering a more rounded than linear approach. Thus, funding bodies (including not only the European Commission but also the Wellcome Trust) had a significant impact in encouraging social science input.

The phenomenon of ‘ELSI-fication’ meant that scholars who had for years been working on ethical, legal, and social issues now had a recognizable ‘brand’, but it was not always regarded with approval by those who preferred to identify with their home discipline and they were sometimes (unfairly) accused of jumping on the latest bandwagon. Throughout the period, some scholars who might be identified by the observer as ‘bioethicists’ regarded themselves as philosophers, legal scholars, or social scientists inspired by the significance of the issues themselves. More recently, the emphasis in European funding has shifted from ELSA to a new acronym: RRI, or ‘responsible research and innovation’. This has been defined by Rene von Schomberg as

a transparent, interactive process by which societal actors and innovators become mutually responsive to each other with a view on the (ethical) acceptability, sustainability and societal desirability of the innovation process and its marketable products (in order to allow a proper embedding of scientific and technological advances in our society).^100^

Ethical acceptability is further explained here as being in compliance with the values of the European Union charter on fundamental rights, such as the right to privacy.^101^

Bioethics has also been subject to a ‘global turn’ thanks to the promotion of a new approach known as ‘global research ethics’ or ‘global bioethics’.^102^ What it might mean for ethics to be ‘global’ is not entirely clear, however. Nigel Dower has drawn a distinction between an ethic that is global in application and an ethic that is global in acceptance.^103^ The first is arguably easier to achieve than the second, given the cultural differences at work in different parts of the world, but even an ethic that is global only in application is challenging. What does it mean for an ethics to apply globally? A further distinction needs to be drawn between extending, say, discussions of the just distribution of healthcare resources from intrastate to the interstate arena on the one hand, and discussing issues that are global *per se* on the other. The latter include issues that of their very nature in principle affect the whole globe, as in the cases of climate change and global pandemics, or in discussions of the human genome as the common heritage of humanity.

In dealing with these issues the question arises as to whether theories of biomedical ethics that have been prominent in the west are adequate in other locations and contexts. Bioethicists have discussed whether Kantianism, utilitarianism, and virtue theory, for instance, can feasibly be applied on a global scale.^104^ These theories, however, were not developed with bioethics specifically in mind, whereas theories that were, such as Beauchamp and Childress’s principles of biomedical ethics, have been canvassed as providing a possible basis for a global bioethics. Raanan Gillon has argued that autonomy, beneficence, non-maleficence and justice are the basis for a global ethics, being universally accepted in some form.^105^ But others have countered by arguing that these principles can be universally accepted because they can be interpreted in different ways, so what appears to be agreement in fact masks profound disagreements. Søren Holm, in addition, has argued that the principlism framework may not travel well and reflects a particularly American perspective.^106^

At the end of the 20th century and at the beginning of the 21st century there was a notable increase in attempts to move away from the dominance of individualistic thinking in bioethics, including the perceived pre-eminence of autonomy in the four principles. One reason for this was the development of biobank research, leading the World Health Organisation, for example, to say that the balance between the individual and collective needed to be rethought. In speaking of genetic databases they said

… the justification for a database is more likely to be grounded in communal value, and less on individual gain … … it leads to the question whether the individual can remain of paramount importance in this context

And

the achievement of optimal advances in the name of the collective good may require a reconsideration of the respective claims so as to achieve an appropriate balance between individual and collective interests, including those of ethnic minorities, from a multi-cultural perspective.^107^

This has been described as a communitarian turn.^108^ This does not mean that individualistic thinking gave way to communitarian thinking, or that bioethicists suddenly became communitarians. It means that principles other than the Georgetown four gained prominence, such as the principle of solidarity, leading to the 2011 report of the Nuffield Council of Bioethics in which solidarity is described as an ‘emerging principle’ in bioethics. It is unusual for the Council to issue reports on particular principles rather than on specific issues. The report explored the ways in which it might be applied.^109^ The relationship between solidarity and justice was also a matter of investigation. The Nuffield Report interpreted solidarity as willingness to bear costs for another’s good, but it is distinct from altruism because of the involvement of reciprocity in a relationship of solidarity. Solidarity comes in different guises. There are distinctions to be drawn between face-to-face and mediated solidarity (as in an insurance company), between the solidarity of a coalition and humanitarian solidarity.^110^

The relationship between the communitarian turn and globalisation needs consideration. On the face of it, solidarity is associated with membership of groups or communities, and so might be thought naturally to combine with exclusion of the interests of those who are not members of the group. The possibility of humanitarian solidarity, where the relevant group includes all human beings, is needed to counteract this potential problem.

The development of feminist bioethics was increasingly influential during the period. Following the establishment of the International Association of Bioethics in the early 1990s, Feminist Approaches to Bioethics^111^ was established as an international network of feminist scholars. The work of feminist bioethicists had considerable impact upon bioethics worldwide. The concerns of feminist bioethics to some extent overlapped with those of communitarians and also extended to global issues. The concept of relational autonomy, for example, emphasized the context of social relationships in which individuals exist, and provided a counter balance to the picture of the autonomous agent as an isolated individual decision maker.^112^ For feminist bioethics power relationships also provided an important focus. In the context of reproductive decision-making, whether concerning genetic testing or termination of pregnancy, the power of women to make a choice in relation to partners, clinicians and the prevailing legal system has to be taken into account. As regards global issues, questions of the global distribution of healthcare resources cannot ignore the stark differentials between men and women in some societies, evident for example in sexual and reproductive health and infant mortality statistics.^113^ These ideas can be found in writers looking at bioethical issues from different perspectives. For example, Onora O’Neill, using a Kantian inspired approach in addressing issues of transnational justice, argued that while we need a system of abstract reasoning, this does not need to be based on the notion of idealized autonomous agents but humans with limited capacities and varied vulnerabilities who interact. Idealized agents have traditionally been based on the model of men and are thus biased in favour of men.^114^

These considerations, in turn, connect feminist, communitarian, and global approaches to the increasing emphasis on public health ethics during the past decade, with issues such as antibiotic resistance, climate change, and obesity now receiving far more attention in bioethics and beyond. But the extent to which this is a new phenomenon is debatable. Justice has always been one of Beauchamp and Childress’s four principles of biomedical ethics,^115^ and questions about the fair distribution of resources, such as organs and dialysis machines, were credited as a major influence behind the emergence of bioethics in the USA during the 1960s and 1970s.^116^ What appears beyond doubt, however, is that the focus on new issues and concerns has fostered a timely re-evaluation of the relationship between individual and collective interests.

## IV. CONCLUSIONS

The ‘turns’ and approaches discussed here represent trends that had the potential to affect the ways in which bioethicists worked, who they worked with, as well as what counted as ‘bioethical’ issues. They stemmed not only from the pre-existing commitments of those individuals and groups who engaged with bioethics, but also in response both to funding initiatives that followed technological developments, such as whole genome sequencing, and to ongoing research assessment initiatives which continue to emphasise the social and economic impact of university research. New approaches are likely to emerge in response to more recent questions surrounding developments such as gene editing, 3-D printing, and biometric technologies, among other issues, but we should be wary of assuming what form they will take, or who will undertake them. By showing how the contours and influence of bioethics are connected to broader social, financial, and political concerns, history reminds us that its status and authority are likely to change in future. Arguably, the current climate appears less conducive for bioethics than at any period in its history. While the possibility and nature of expertise in bioethics has long been an issue, claims that ‘red tape’ are today stifling innovation and a distrust of ‘experts’ in multiple sectors threatens to undermine the goodwill which doctors and politicians showed towards bioethicists in the 1980s and 1990s.^117^ At the same time, some bioethicists worry that the academic centres they helped establish face a diminished student intake and an uncertain future, with undergraduate tuition fees of £9,000 per year (at the time of writing) and the ongoing focus on research performance evaluation raising the prospect of universities attaching ‘less value to the taught postgraduate courses that have educated so many health professionals in ethics’.^118^ The lawyers, philosophers, and social scientists who look to engage with bioethics in years to come cannot presume their input will be welcomed or even deemed necessary, and may have to find new ways of asserting why it benefits doctors, scientists, and the public at large.

## Footnotes

1 Jahr’s work went largely unnoticed during his lifetime, but several authors have recently analysed it as part of the longer ‘pre-history’ of bioethics and as a possible bridge between bioethics and environmental ethics. See F Steger, ‘Fritz Jahr’s (1895-1953) European Concept of Bioethics and Its Application Potential’ (2015) 6 J European Journal of Bioethics JAHR 215; HM Sass, ‘Fritz Jahr’s 1927 Concept of Bioethics’ (2008) 17 Kennedy Institute of Ethics Journal 279.

2 W Reich, ‘The Word “Bioethics”: Its Birth and the Legacies of Those Who Shaped It’ (1994) 4 Kennedy Institute of Ethics Journal 279; VR Potter, ‘Bioethics, The Science of Survival’, 14 Perspectives in Biology and Medicine 127.

3 Potter, ibid 130.

4 Reich (n 2) 325.

5 ibid 330–31; D Callahan, ‘Bioethics as a Discipline’ (1973) 1 Hastings Center Studies 66.

6 For more background, see R Baker, *Before Bioethics: A History of American Medical Ethics from the Colonial Period to the Bioethics Revolution* (OUP 2013).

7 On how legal and theological discussion of abortion consolidated medical authority in this period, see A Jonsen, *A Short History of Medical Ethics* (OUP 2013) 77–79.

8 Callahan (n 5) 71.

9 D Callahan, ‘The Hastings Center and the Early Years of Bioethics’ (1999) 9 Kennedy Institute of Ethics Journal 53.

10 Quoted in D Rothman, *Strangers at the Bedside* (Basic Books 1991) 188.

11 R Fox and J Swazey, *Observing Bioethics* (OUP 2008) 128–45.

12 JH Evans, ‘A Sociological Account of the Growth of Principalism’ (2000) 30 Hastings Center Report 31.

13 Rothman, *Strangers at the Bedside* (1991) 189.

14 S Jasanoff, *Designs on Nature: Science and Democracy in Europe and the United States* (Princeton UP 2014); Fox and Swazey (n 11) 215–85; F Moazam and AM Jarafey, ‘Pakistan and Biomedical Ethics: Report from a Muslim Country’ (2005) 14 Cambridge Quarterly of Healthcare Ethics 249; ML Gross and V Ravitsky, ‘Israel: Bioethics in a Jewish-Democratic State’ (2003) 12 Cambridge Quarterly of Healthcare Ethics 247; DRJ Macer, ‘Bioethics in Japan and East Asia’ (2001) 9 Turkish Journal of Medical Ethics 70.

15 D Reubi, ‘The Will to Modernize: A Genealogy of Biomedical Research Ethics in Singapore’ (2010) 4 International Political Sociology 142.

16 M Dixon-Woods and R Ashcroft, ‘Regulation and the Social Licence for Medical Research’ (2008) 11 Medical Health Care and Philosophy 381; B Culliton and WK Waterfall, ‘The Flowering of American Bioethics’ (1978) 2 British Medical Journal 1270.

17 D Wilson, *The Making of British Bioethics* (Manchester UP, 2014) 257.

18 I Kennedy, *Unmasking Medicine* (Allen and Unwin 1981) xi; M Warnock, ‘Moral Thinking and Government Policy: The Warnock Committee on Human Embryology’ (1985) 63 Millbank Memorial Fund Quarterly. Health and Society 514.

19 M Thomas, ‘Should the Public Decide?’ (1981) 7 Journal of Medical Ethics 182; Anon, ‘Research Ethics Committees’ (1984) 321 The Lancet 1026.

20 O O’Neill, *Autonomy and Trust in Bioethics* (OUP 2001) 1.

21 Wilson (n 17) 171, 257.

22 L Back, *Academic Diary: Or Why Higher Education Still Matters* (Goldsmiths Press 2016) 8; B Readings, *The University in Ruins* (Harvard UP 1996).

23 H Perkin, *The Rise of Professional Society: England Since 1800* (Routledge 1990) 4.

24 JV Pickstone, ‘Thomas Percival and the Production of Medical Ethics’ in R Baker, D Porter and R Porter (eds), *The Codification of Medical Morality: Historical and Philosophical Studies on the Formalization of Western Medical Morality in the Eighteenth and Nineteenth Centuries* (Kluwer Academic 1993) 161.

25 M Brown, *Performing Medicine* (Manchester UP 2014) 226.

26 P Weindling, ‘Human Guinea Pigs and the Ethics of Experimentation on Man: The BMJ’s Correspondent at the Nuremberg Medical Trial’ (1996) 313 British Medical Journal 1467.

27 R Bud, ‘Penicillin and the New Elizabethans’ (1998) 31 British Journal for the History of Science 305.

28 A Abbott, *The System of Professions: An Essay on the Division of Expert Labour* (University of Chicago Press 1988) 2.

29 M Davies, *Textbook on Medical Law* (2nd edn, Blackstone Press 1998) 94.

30 Denning J, quoted in, ibid 95.

31
*Bolam v Friern Hospital Management Committee* [1957] 2 All ER 118.

32 [2015] UKSC 11.

33 SW Chan and others, ‘Montgomery and Informed Consent: Where are We Now?’ (2017) 357 British Medical Journal 2224. For more detail on how the courts have recently diluted or rejected the ‘*Bolam*’ test see M Brazier and E Cave, *Medicine, Patients and the Law* (6th edn, Manchester UP 2016) 201–09.

34 M Brazier, *Medicine, Patients and the Law* (3rd edn, Penguin 2003) 102.

35 A MacIntyre, *A Short History of Ethics* (Routledge Classics 2002) 242–45; see also B Rogers, *AJ Ayer: A Life* (Chatto and Windus 1999) 48.

36 AJ Ayer, *Language, Truth and Logic* (Penguin 2001) 99.

37 N Krishna, ‘Is Goodness Natural?’ *Aeon Magazine* <aeon.co/amp/essays/how-philippa-foot-set-her-mind-against-prevailing-moral-philosophy> accessed on 14 December 2017.

38 Ayer (n 36) 99.

39 L Wittgenstein, quoted in C Waddington, *Science and Ethics* (Unwin Brothers 1942) 7.

40 ibid, 26–29.

41 MH Pappworth, *Human Guinea Pigs: Experiments on Man* (Routledge and Kegan Paul 1967) 204.

42 ibid 208.

43 Ministry of Health statement, quoted in A Hedgecoe, ‘A Form of Practical Machinery: The Origin of Research Ethics Committees in the United Kingdom’ (2009) 53 Medical History 331.

44 C Clothier to M Pappworth (29 March 1978) held at Wellcome Trust Library for History of Medicine, Archives and Manuscripts, PP/MHP/C/5.

45 ibid.

46 Wilson (n 17) 71–73; CG Brown, *The Death of Christian Britain: Understanding Secularisation, 1800–2000* (Routledge 2001).

47 I Ramsey, ‘A New Prospect in Theological Studies’ (1964) 67 Theology 532.

48 I Ramsey, ‘Introduction’ (1968) typescript notes held at the Durham Cathedral archives.

49 I Ramsey, ‘Moral Problems Facing the Medical Profession at the Present Time’, Inaugural Address to British Medical Association Annual Clinical Meeting (1972) held at Durham Cathedral archives.

50 A Campbell, ‘Philosophy and Medical Ethics’ (1976) 2 Journal of Medical Ethics 1.

51 I. Kennedy (n 18) vii.

52 I Kennedy, ‘The Patient on the Clapham Omnibus’ (1984) 47 Modern Law Review 454.

53 Wilson (n 17) 114.

54 ibid; I Kennedy, ‘The Karen Quinlan Case: Problems and Proposals’ (1976) 7 Journal of Medical Ethics 3.

55 ibid.

56 I Kennedy, ‘What is a Medical Decision?’ in I Kennedy, *Treat Me Right: Essays in Medical Law and Ethics* (Clarendon Press 1988) 27. This chapter is text of the Astor Memorial Lecture that Kennedy gave at the Middlesex Hospital, London, in July 1979.

57 I Kennedy, ‘Consumerism in the Doctor-Patient Relationship’ *Listener* (11 December 1980) 777.

58 I Kennedy, ‘Medical Ethics are not Separate from but Part of Other Ethics’ *Listener* (27 November 1980) 715.

59 K Boyd, ‘The Discourses of Bioethics in the United Kingdom’ in RB Baker and LB McCullough (eds), *The World History of Medical Ethics* (CUP 2009) 486.

60 J Katz, *Experimentation with Human Beings* (Russell Sage Foundation 1972) 5.

61 I Kennedy, ‘Response to the Critics’ (1981) 7 Journal of Medical Ethics 202.

62 ibid.

63 I Kennedy, *The Unmasking of Medicine* (Paladin Press 1983) 5.

64 E Ackroyd, ‘Mr Kennedy and Consumerism’ (1981) 7 Journal of Medical Ethics 180.

65 Wilson (n 17) 120–22; Perkin (n 23) 472–80. See also N Lawson, *The New Conservatism* (Centre for Policy Studies, 1980).

66 Kennedy, ‘Preface’ in *Treat Me Right: Essays in Medical Law and Ethics* (Clarendon Press 1988) viii.

67 JD Swales, ‘Thoughts on the Reith Lectures’ (1981) 7 Journal of Medical Ethics 1348.

68 D Black, ‘Both Sides of a Public Face’ (1981) 202 British Medical Journal 2044.

69 On criticism of IVF and embryo research in the early 1980s, see Wilson (n 17) 152–55.

70 ibid 157.

71 Kennedy (n 61) 152.

72 M Warnock, ‘Scientific Research Must Have a Moral Basis’ *New Scientist* (15 November 1984) 36.

73 Warnock (n 18) 514.

74 M Warnock, *People and Places: A Memoir* (Duckworth 2000) 49; ibid, *Ethics Since 1900* (OUP 1960) 203.

75 ibid 204.

76 P Foot, ‘Moral Arguments’ (1958) 67 *Mind* 502.

77 Warnock (n 74) 206.

78 See the essays in P Singer (ed), *Applied Ethics* (OUP 1986).

79 M Warnock, *Ethics Since 1900* (3rd edn, OUP 1978) 139.

80 Singer (n 78) 4.

81 P Scott, *The Crisis of the University* (Croom Helm 1984).

82 D Austin, ‘A Memoir’ (1982) 17 Government and Opposition 469.

83 B Pullan and M Abendstern, *A History of the University of Manchester, 1973– 1990* (Manchester UP 2004) 249.

84 ibid 243.

85 Kennedy (n 61) 5.

86 D Pond (chair) *Teaching of Medical Ethics* (Institute of Medical Ethics) 51; see also General Medical Council, *Tomorrow’s Doctors: Recommendations for Undergraduate Medical Education* (General Medical Council 1993).

87 Wilson (n 17) 201.

88 S Toulmin, ‘How Medicine Saved the Life of Ethics’ (1982) 25 Perspectives in Biology and Medicine 736.

89 R Gillon, ‘Defending “The Four Principles” Approach to Biomedical Ethics’ (1995) 21 Journal of Medical Ethics 323; Evans (n 12).

90 Wilson (n 17) 167–71; 194–96; D Archard, ‘Why Philosophers Are Not and Should Not be Moral Experts’ (2011) 25 Bioethics 119.

91 J Harris and H Lesser, ‘Moral Philosophy’ (held at the archives for the Centre for Social Ethics and Policy, University of Manchester 1987).

92 R Fox and J Swazey, ‘Medical Morality is not Bioethics – Medical Ethics in China and the United States’ (1984) 27 Perspectives in Biology and Medicine 336.

93 ibid 337. See also R de Vries and others (eds), *The View from Here: Bioethics and the Social Sciences* (Blackwell Publishing 2007) 12.

94 A Hedgecoe, ‘Critical Bioethics: Beyond the Social Science Critique of Applied Ethics’ (2004) 18 Bioethics 120; E Haimes, ‘What Can the Social Sciences Contribute to the Study of Ethics?’ (2002) 16 Bioethics 89.

95 P Borry, P Schotsmans and K Dierickx, ‘The Birth of the Empirical Turn in Bioethics’ (2005) 19 Bioethics 49.

96 De Vries and others (n 93) 2–3.

97 See for example, T Bracanovic, ‘From Integrative Bioethics to Pseudoscience’ (2012) 12 Developing World Bioethics 148–56.

98 AC Molewijk and others, ‘Implicit Normativity in Evidence-based Medicine: a Plea for Integrated Empirical Ethics Research (2003) 11 Health Care Analysis 69–92.

99 R Chadwick and M Levitt ‘Complementarity: Multidisciplinary Research in Bioethics’ in S Gindro, R Bracalenti and E Mordini (eds), *Bioethics Research: Policy, Methods and Strategies* (European Commission, Belgium: Report EUR17465EN, 1997) 73–82.

100 R von Schomberg, Introduction in *Towards Responsible Research and Innovation in the Information and Communication Technologies and Security Technologies Fields* (2011) 9.

101 ibid.

102 See for example, RM Green, A Donovan and SA Jauss (eds), *Global Bioethics: Issues of Conscience for the Twenty-First Century* (Clarendon Press 2008).

103 N Dower, Global Ethics, Approaches. In R Chadwick and others, *Encyclopedia of Applied Ethics* (2nd edn, Elsevier 2012) 504–13.

104 R Chadwick, and A O’Connor, ‘Ethical Theory and Global Challenges’ in D Moellendorf and H Widdows (eds), *The Routledge Handbook of Global Ethics* (Routledge 2015) 24–34.

105 R Gillon, ‘Ethics Needs Principles – Four Can Encompass the Rest – and Respect for Autonomy Should Be “First Among Equals”’ (2003) 29 Journal of Medical Ethics 307–12; ‘Medical ethics: four principles plus attention to scope’ (1994) 309 British Medical Journal 184–88.

106 S Holm, ‘Not Just Autonomy – the Principles of American Biomedical Ethics’ (1995) 21 Journal of Medical Ethics 332–38.

107 World Health Organization, *Genetic Databases: Assessing the Benefits and the Impact on Human and Patient Rights* (WHO 2003).

108 R Chadwick ‘The Communitarian Turn – Myth or Reaity?’ (2009) Cambridge Quarterly of Healthcare Ethics 155–65.

109 B Prainsack and A Buyx *Solidarity: an emerging principle in Bioethics* (Nuffield Council of Bioethics 2011).

110 R ter Meulen, ‘Solidarity and Justice in Health Care: a Critical Analysis Of Their Relationship’ (2015) 43 Diametros 1–20.

111 <Fabnet.org> accessed 14 December 2017.

112 C Mackenzie and N Stoljar, (eds), *Relational Autonomy: Feminist Perspectives on Autonomy, Agency and the Social Self* (OUP 2000).

113 See for example, A Donchin and S Dodds, *Linking Visions: Feminist Bioethics and the Developing World* (Rowman and Littlefield 2004); also the website of the Feminist Approaches to Bioethics network www.fabnet.org.

114 O O’Neill ‘Justice, Gender and International Boundaries’ British Journal of Political Science 20(4) 439–59; *Bounds of Justice* (CUP 2000).

115 TL Beauchamp and JF Childress, *Principles of Biomedical Ethics* (7th edn, OUP 2013).

116 D Adams, ‘Artificial Kidneys and the Emergence of Bioethics: The History of “Outsiders” in the Allocation of Haemodialysis’ (2010) 24 Social History of Medicine 461.

117 Wilson (n 17) 239–43.

118 M Brazier, R Gillon and J Harris, ‘Helping Doctors Become Better Doctors: Mary Lobjoit – An Unsung Heroine of Medical Ethics’ (2012) 38 Journal of Medical Ethics 383.

